# A randomized, double-blind, placebo-controlled, parallel-group study of once-daily inhaled fluticasone furoate on the hypothalamic–pituitary–adrenocortical axis of children with asthma

**DOI:** 10.1186/s13223-020-0406-6

**Published:** 2020-02-04

**Authors:** Philippe Bareille, Susan Tomkins, Varsha Imber, Mohammed Tayob, Karen Dunn, Rashmi Mehta, Sanjeev Khindri

**Affiliations:** 1 Medicines Research Centre, GlaxoSmithKline, Gunnels Wood Rd, Stevenage, SG1 2NY Hertfordshire UK; 2 GlaxoSmithKline, Uxbridge, Middlesex UK; 3 Mzansi Ethical Research Centre, Cape Town, South Africa; 4 North Carolina Clinical Research, Raleigh, NC USA; 5 GlaxoSmithKline Research Triangle Park, Durham, NC USA

**Keywords:** Asthma, Pediatric, Fluticasone furoate (FF), Safety, HPA axis, Serum cortisol

## Abstract

**Background:**

To evaluate the effects of fluticasone furoate on the hypothalamic–pituitary–adrenocortical axis, and the safety and tolerability of fluticasone furoate treatment in children with asthma.

**Methods:**

This was a randomized, double-blind, placebo-controlled, multicenter, stratified, parallel-group, non-inferiority study of fluticasone furoate 50 µg inhalation powder administered once daily. The study enrolled children (aged 5–11 years inclusive) with a documented diagnosis of asthma for ≥ 6 months and a Childhood Asthma Control Test score of > 19. After a 7–14-day run-in period, eligible subjects were stratified by age and randomized to fluticasone furoate 50 µg once daily or placebo once daily via ELLIPTA for 6 weeks. The primary endpoint was the change from baseline (expressed as a ratio) in 0–24-h weighted mean serum cortisol at the end of the treatment period.

**Results:**

Fifty-six randomized subjects received fluticasone furoate 50 µg once daily and 55 received placebo. The primary analysis was performed in the serum cortisol population (n = 104) and demonstrated that fluticasone furoate 50 µg once daily was non-inferior to placebo (ratio = 0.93; 95% confidence interval 0.8096, 1.0620), as the lower limit of the 95% confidence interval for the geometric mean treatment ratio of fluticasone furoate 50 µg once daily versus placebo was greater than 0.80. Findings from the intent-to-treat population (n = 111) were similar.

**Conclusions:**

Six weeks of treatment with inhaled fluticasone furoate 50 µg once daily had no clinically relevant effect on the hypothalamic–pituitary–adrenocortical axis function of children, as measured by 24-h serum cortisol profiles. The primary analysis showed that fluticasone furoate 50 µg once daily was non-inferior to placebo. Fluticasone furoate 50 µg once daily was well tolerated and no new safety concerns emerged during the study.

**Trial registration:**

This study is registered in ClinicalTrials.gov (NCT02483975). Date of submission: 25 June 2015.

## Background

Inhaled corticosteroids (ICS) are a mainstay of treatment in adults and children with persistent asthma of varying severity [1]. ICS are recommended by the Global Initiative for Asthma (GINA) guidelines in children aged 6–11 years with persistent asthma that is not sufficiently controlled by a short-acting β_2_-agonist (SABA) [1].

Although ICS are effective anti-inflammatory treatments of persistent asthma [1], their excessive or long-term use at high dosages is associated with hypothalamic–pituitary–adrenocortical (HPA) axis suppression in adults and children, potentially leading to adrenal insufficiency and in severe cases, adrenal crisis and death [2–4].

Fluticasone furoate (FF), delivered via the ELLIPTA inhaler (GlaxoSmithKline, Middlesex, UK), is an ICS that has been approved as a once-daily (QD) maintenance treatment for asthma prophylaxis in patients ≥ 5 years of age in the USA [5].

The safety of inhaled FF in combination with the long-acting β_2_-agonist vilanterol (VI) on the HPA axis in adult and adolescent (12–17 years of age) patients with asthma has been established [6]. A phase 2b, dose–response, efficacy and safety study showed that FF was effective and well tolerated in children aged 5–11 years with uncontrolled asthma [7]. Two randomized, double-blind, crossover studies of FF 100 µg or FF/VI 100/25 µg found no evidence of serum cortisol (SC) suppression in children (aged 5–11 years) with persistent asthma, suggesting this FF dose does not affect the HPA axis in this patient population [8, 9].

Nevertheless, data on the effects of FF on HPA axis suppression in children are limited. The primary objective of this study was to evaluate the effect of 6 weeks of treatment with QD inhaled FF 50 µg (FF 50 QD) on the HPA axis in children aged 5–11 years compared with placebo. Secondary objectives included safety and tolerability assessments and to determine the FF plasma concentration at 30 min post-dose after 6 weeks of treatment with FF 50 QD.

## Methods

### Study design

**Fig. 1:**
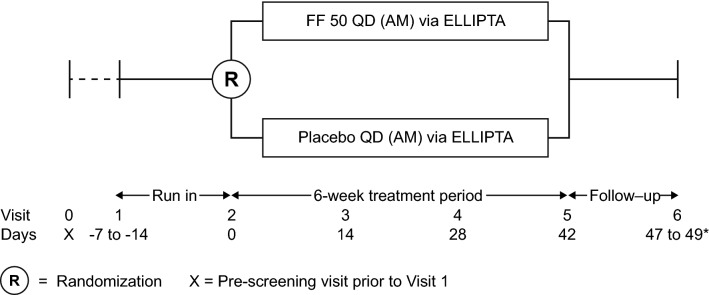
Study design. Subjects received open-label montelukast during the run-in and treatment periods, and albuterol/salbutamol as needed to treat acute asthma symptoms. *Days 5–7 after the end of treatment. *FF 50 QD* fluticasone furoate 50 µg once daily

This was a randomized, double-blind, placebo-controlled, stratified, parallel-group, non-inferiority study (Fig. 1), conducted at 13 clinics in the USA and three clinics in South Africa.

After a 7–14-day run-in period, eligible subjects were stratified by age (5–< 8 and 8–< 12 years) and randomized to FF 50 QD or placebo QD via ELLIPTA for 42 days (6 weeks; treatment period). The first dose of study drug was administered in the clinic under supervision at Visit 2 (randomization) after 24-h serum sampling and urine collection. Throughout the run-in and treatment periods, subjects received open-label montelukast. Albuterol/salbutamol was provided to use on an as-needed basis to treat acute asthma symptoms. Serum sampling and urine collection (both over the 24-h period) were repeated at Visit 5 (end of treatment). Blood samples were also collected to determine FF plasma levels at Visit 5 (pre-dose and 30 min post-dose).

The study protocol was approved by an ethics committee or institutional review board at each study site and conducted in accordance with the International Conference for Harmonisation of Technical Requirements for Registration of Pharmaceuticals for Human Use (ICH) Good Clinical Practice (GCP) and applicable country-specific requirements, and the ethical principles of the Declaration of Helsinki. Protocol amendments after study initiation included clarifying the lower reference range and removing the upper reference range for the randomization exclusion criterion for SC. Informed written consent was obtained from at least one parent/caregiver along with assent from the subject. This study is registered in ClinicalTrials.gov (NCT02483975). Anonymized individual participant data and study documents can be requested for further research from http://www.clinicalstudydatarequest.com.

### Study population

Eligible children were males and premenarchal females, aged 5–11 years (inclusive) and weighing ≥ 17 kg, with a documented diagnosis of asthma for at least 6 months prior to Visit 1 (run-in period) and a Childhood Asthma Control Test score of > 19. They had been receiving SABA alone or in combination with non-corticosteroid controller medication(s) for asthma management for at least 4 weeks prior to Visit 1. Exclusion criteria are provided in the online only content (Additional file 1: Methods S1).

Subjects were assigned to FF 50 QD or placebo at Visit 2 in accordance with a randomization schedule generated prior to study start using validated internal software. Randomization schedules were concealed until database freeze was achieved. Children were eligible for randomization if they generated a peak expiratory flow > 75% of their personal best measured in the last 7 days of the run-in period and completed 24-h SC assessments and a 24-h urine collection prior to randomization at Visit 2. Further details on treatment eligibility and criteria for withdrawal are provided in the online only content (Additional file 1: Methods S1).

### Endpoints

The primary endpoint was the change from baseline (expressed as a ratio) in 0–24 h weighted mean SC at the end of the treatment period. Secondary endpoints were the change from baseline (expressed as a ratio) at the end of the treatment period in: area under the curve 0–24-h (AUC_0–24_) SC; 24-h urinary cortisol (UC) excretion; and 24-h 6-β hydroxycortisol excretion. Other endpoints included the change from baseline in trough SC at the end of the 6-week treatment period and FF plasma concentrations. The incidence of adverse events (AEs) was monitored throughout the study.

### Pharmacodynamic assessments

Samples for SC and UC (free cortisol and 6-β hydroxycortisol) analysis were taken over 24-h periods at Visit 2 (start time between 7:00 am and 9:00 am) and Visit 5 (start time before 10:00 am). For SC, 2 mL blood samples were taken at 0, 2, 4, 8, 12, 16, and 24-h time points relative to sampling start time (24-h sampling was completed prior to administration of first dose at Visit 2; sampling was post-dose at Visit 5). SC was measured via an approved, validated high-performance liquid chromatography (HPLC) system in combination with a tandem mass spectrometer (MS/MS). The lower limit of quantification (LLQ) of the assay was 1 μg/dL (28 nmol/L). Further details of SC and UC assays are provided in the online only content (Additional file 1: Methods S1).

### Pharmacokinetic assessments

Blood samples (2 mL) were taken for pharmacokinetic (PK) analysis at Visit 5, pre-dose and at 0.5-h post-dose. Plasma samples were analyzed for FF using solid phase extraction followed by HPLC–MS/MS analysis with the LLQ for FF 10 pg/mL.

### Populations and statistical analyses

The intent-to-treat (ITT) population comprised all subjects randomized to treatment and who received at least one dose of study drug and was the primary population for the safety analyses. The SC population included all subjects in the ITT population with no protocol violations considered to affect the SC endpoint and whose serum samples were not considered to have confounding factors that would affect interpretation of the results. The decision to exclude a subject from the SC population was made prior to unblinding of the study. Analysis of the primary endpoint was based on the SC population, with the ITT population used for supportive analyses (with equal weighting). The UC population included all subjects who had no protocol violations that were considered to affect the UC endpoint and was the primary population for UC analyses. This population was also determined prior to unblinding of the study. The PK population comprised subjects in the ITT population for whom a PK sample was obtained and analyzed.

The SC weighted mean (0–24-h) was calculated by dividing the AUC_0–24_ by the time period. The ratio from baseline of the 0–24-h weighted mean SC concentration was log_e_ transformed prior to analysis. The log_e_ transformed ratio was compared between treatment groups (SC and ITT populations) using an analysis of covariance (ANCOVA) model, allowing for the effects of baseline (log_e_ transformed), age, and sex. Treatment ratios for each comparison were calculated by back-transforming the difference between the least square means. Using the pooled estimate of variance, the 95% confidence interval (CI) was calculated for the difference and then back-transformed. Non-inferiority would be demonstrated if the lower limit of the (geometric mean ratio of FF 50 QD and placebo) two-sided 95% CI was greater than 0.80. Further information on study populations and statistical analyses is provided in the online only content (Additional file 1: Methods S1).

## Results

### Demographics and baseline characteristics

Of 156 subjects screened, 111 were randomized and received study drug (FF 50 QD, n = 56; placebo, n = 55; ITT population). Of these, 107 (96%) subjects completed the study (between 9 October, 2015 and 21 June, 2016) (Additional file 2: Figure S1), 104 (94%) subjects were included in the SC population, 108 (97%) subjects were included in the UC population and 106 (95%) were included in the PK population. Four (4%) subjects withdrew prematurely from the study treatment, primarily due to being lost to follow-up. Seven subjects were excluded from the SC population due to missing 24-h SC concentrations (n = 4) or study treatment compliance deviations (n = 3). Three subjects were excluded from the UC population owing to study treatment compliance deviations.

Demographics and baseline characteristics were generally comparable between groups (Table 1) and were similar between the SC and ITT populations. The mean age of the subjects was 8.4 years and 55% of the subjects were male (Table 1).

**Table 1:** Demographics and baseline characteristics (ITT population)

Demographic characteristic	Placebo group N = 55	FF 50 QD group N = 56	Total N = 111
Age (years)			
Mean (SD)	8.4 (2.00)	8.4 (1.97)	8.4 (1.97)
Min., Max.	5, 11	5, 11	5, 11
Age group (years)			
5–< 8	19 (35)	20 (36)	39 (35)
8–< 12	36 (65)	36 (64)	72 (65)
Sex			
Female	30 (55)	20 (36)	50 (45)
Male	25 (45)	36 (64)	61 (55)
Height (cm)			
Mean (SD)	134.6 (14.37)	135.3 (15.10)	135.0 (14.68)
Min., Max.	106, 161	100, 173	100, 173
Weight (kg)			
Mean (SD)	35.88 (14.338)	35.83 (15.054)	35.86 (14.637)
Min., Max.	18.5, 71.5	17.2, 87.7	17.2, 87.7

Values are n (%), unless otherwise stated, where n represents the number of subjects

*FF 50 QD* fluticasone furoate 50 µg once daily, *ITT* intent-to-treat, *Min.* minimum, *Max.* maximum, *SD* standard deviation

### Exposure and treatment compliance

The mean exposure to study treatment (ITT population) was similar between groups (placebo: 42.4 days; FF 50 QD: 40.9 days). Most subjects received study treatment for > 35–≤ 42 days (FF 50 QD: 61%; placebo: 55%); the majority of the remainder of subjects were exposed to study treatment for > 42 days (FF 50 QD: 36%; placebo: 44%).

Mean compliance (according to the dose counter on the inhaler device; ITT population), was comparable between groups: placebo 106.7%; FF 50 QD 103.0%. Most subjects were within the compliance range of ≥ 80–≤ 120%, with only one subject in the FF 50 QD group demonstrating compliance below 80%.

### Pharmacodynamics

#### Primary analysis

**Fig. 2:**
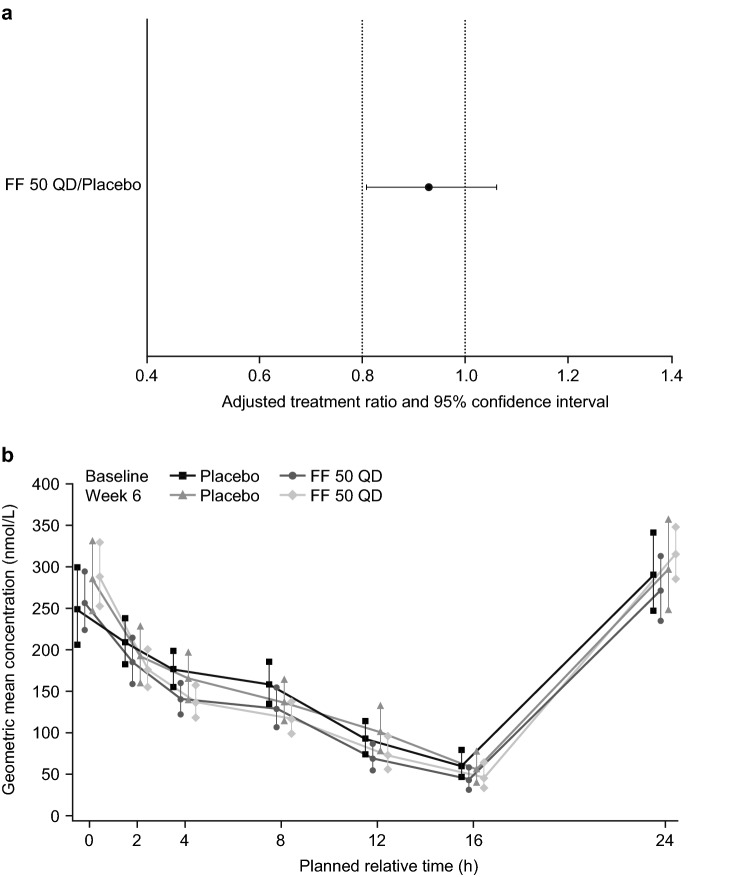
**a** Adjusted treatment ratios for SC weighted mean (0–24 h) at week 6; **b** geometric mean (95% CI) SC concentration–time profile by treatment group and visit (SC population). *CI* confidence interval, *FF 50 QD* fluticasone furoate 50 µg once daily, *h* hours, *SC* serum cortisol

At week 6, the geometric mean for the derived SC weighted mean (0–24-h) was similar to the respective baseline values in both treatment groups (FF 50 QD group: baseline, 153.20 nmol/L and week 6, 157.52 nmol/L; placebo group: baseline, 177.22 nmol/L and week 6, 176.83 nmol/L; Additional file 3: Table S1). Analysis of the SC weighted mean (0–24-h) in the SC population demonstrated that FF 50 QD was non-inferior to placebo (ratio = 0.93 [95% CI 0.8096, 1.0620]; Table 2) since the lower limit of the 95% CI for the geometric mean treatment ratio of FF 50 QD versus placebo was greater than 0.80 (Fig. 2a). Findings from the ITT population were similar to those for the SC population and showed non-inferiority of FF 50 QD versus placebo (Table 2 and Additional file 3: Table S1). The geometric mean SC concentration—24-h time profiles were similar between treatment groups at baseline and week 6 (Fig. 2b).

**Table 2:** Change from baseline (expressed as a ratio) in 24-h weighted mean SC at week 6

Variables	Placebo group	FF 50 QD group
SC population		
N	51	53
n	50	52
LS geometric mean (nmol/L)	173.25	160.65
LS ratio to baseline	1.05	0.97
FF 50 QD/placebo ratio	–	0.93
95% CI	–	0.8096, 1.0620
ITT population		
N	55	56
n	51	53
LS geometric mean (nmol/L)	174.08	159.98
LS ratio to baseline	1.06	0.97
FF 50 QD/placebo ratio	–	0.92
95% CI	–	0.8040, 1.0505

*CI* confidence interval, *FF 50 QD* fluticasone furoate 50 µg once daily, *h* hours, *ITT* intent-to-treat, *LS* least squares, *SC* serum cortisol, *N* number of subjects in population, *n* number of subjects with value at the visit

#### Secondary endpoints

The geometric mean SC AUC_0–24_ was similar at baseline and week 6 within both treatment groups (Additional file 3: Table S1). Based on the statistical analysis, the ratio for the change from baseline in SC AUC_0–24_ for FF 50 QD over placebo was 0.93 (95% CI 0.81, 1.06).

The geometric mean 24-h UC excretion and 6-β hydroxycortisol excretion (geometric mean) were also similar at baseline and week 6 within both treatment groups (Additional file 3: Table S1). The ratio of 24-h UC excretion for FF 50 QD over placebo was 0.79 (95% CI 0.61, 1.03) and that of 6-β hydroxycortisol excretion for FF 50 QD over placebo was 0.88 (95% CI 0.72, 1.07).

#### Other endpoints

At week 6, the geometric mean SC trough drug (24-h planned time point) was 315.51 nmol/L for the FF 50 QD group and 296.64 nmol/L for the placebo group (Additional file 4: Table S2). Based on the statistical analysis, the ratio of SC trough drug (24-h) for FF 50 QD over placebo from baseline was 1.10 (95% CI 0.90, 1.34).

A post hoc summary of the peak SC values observed over the 24-h period at baseline and week 6 determined the ratio for the change from baseline to be 1.03 in the FF 50 QD group and 1.10 in the placebo group (Additional file 4: Table S2).

### Pharmacokinetics

Fifty-three subjects provided blood samples, with data available for 52 subjects at Visit 5. Plasma concentrations of FF were quantifiable in 4/52 subjects pre-dose and 35/52 subjects at 30 min post-dose. The median FF concentration was 12.05 pg/mL at 30 min post-dose (t_max_; time to peak plasma concentration) and ranged from 0 to 41.7 pg/mL with a variance of 88%.

### Safety

The proportion of subjects experiencing an AE (on- or post-treatment) in the FF 50 QD and placebo groups was 13% and 18%, respectively. While on treatment, the most commonly reported AEs were infections and infestations (7% of subjects in each group), followed by gastrointestinal disorders (no cases in the FF 50 QD group and 7% of subjects in placebo group). One case of tonsillitis (post-treatment) was reported in the FF 50 QD group. Only one drug-related AE was reported (gastritis; placebo group), and no serious AEs (SAEs) were reported during the study.

Three AEs of special interest were reported: one case of oropharyngeal pain in the FF 50 QD group and two cases (of oropharyngeal pain and allergic sinusitis, respectively) in the placebo group; all were deemed unrelated to the study drug.

## Discussion

While ICS are established as an effective anti-inflammatory treatment for all severities of persistent asthma [1], their use is associated with a dose-related risk of HPA axis suppression [10, 11]. Although rare, HPA axis suppression may occur in children taking standard doses of corticosteroids [11, 12]. Diminished cortisol secretion by the adrenal cortex is an early sign of adrenal suppression and can occur within hours or days of high-dose corticosteroid administration [13–15]. Loss of the effects of adrenocorticotropic hormone (ACTH) on the adrenal gland in response to chronic use of exogenous corticosteroids results in adrenal atrophy and loss of function [16]. Evaluation of endogenous cortisol secretions is complicated due to the presence of circadian rhythms, with peak concentrations in the morning and trough concentrations around midnight [17]. Therefore, single time point measures are considered insensitive measures of HPA axis function [18]. The 24-h SC concentration time curve is the most sensitive method for detecting changes in cortisol production and diurnal variation after taking exogenous glucocorticoids [18].

The current study investigated the effects of FF inhalation powder on the HPA axis system of children, assessed through the pharmacodynamics (PD) of cortisol secretion. No clinical decrease in derived weighted mean SC was observed from baseline to week 6 in the FF 50 QD group; FF 50 QD was also non-inferior when compared with placebo. A suppressive effect on the HPA axis would have led to reduced cortisol secretion; therefore, the high peak SC values observed in a post hoc summary in this study suggest normal adrenal function in children undergoing treatment with FF 50 QD. Although not evaluated in this study, a potential suppressive effect of long-term administration of FF 100 QD on the HPA axis cannot be ruled out; however, this dose is not currently indicated for use in children aged 5–11 years [5].

The risk of adrenal insufficiency was shown in previous studies to increase with ICS dose and treatment duration [10], as the adrenal gland needs prolonged suppression (i.e., several weeks or months) before losing functionality [18]. The use of a standard ICS dose in the present study and the 6-week treatment period mean that the adrenal response to stress was likely unaffected, even though this does not exclude the risk of long-term adrenal failure after a normal ACTH test. However, given the sensitivity of cortisol secretion to exogenous steroids, the finding of undiminished cortisol secretion after 6 weeks of FF 50 QD is reassuring and suggests that the risk of adverse adrenal effects may not increase with long-term use of FF. However, it is acknowledged that there may be inter-individual variations in the sensitivity of the adrenal gland; this is a well-established class effect, and thus it is recommended that one should remain vigilant, especially in children receiving long-term treatment.

A wealth of recent data has shown that prolonged use (several months or years) of even low-to-medium ICS doses can lead to adrenal suppression [19–22]. The assessment of adrenal insufficiency was usually based on a single point morning cortisol or ACTH test [20–22]. As noted above, a single morning cortisol test is not considered sufficiently sensitive for measuring HPA axis function. In addition, some subjects may have taken oral corticosteroids (OCS), thus confounding the results. It is also possible that a minority of more susceptible individuals experienced an early suppressive effect. To our knowledge, there are no published studies showing the 24-h cortisol profiles of subjects with a documented normal profile after short-term ICS who underwent long-term treatment. Therefore, it is not clear whether the HPA axis response to low-dose ICS can become more sensitive over time, leading to loss of functionality.

In the current study, plasma FF concentrations were measured using pre-dose and 30 min post-dose blood samples, since the median t_max_ following inhaled administration of FF or FF/VI is around 0.5 h [5, 23]. An examination of the maximum concentration of FF achieved after dosing (C_max_) from other pediatric studies [7–9] indicated that the average C_max_ obtained in the current study (~ 12 pg/mL) was dose proportional with the values reported in the literature. The median C_max_ observed in this study was also consistent with the predicted average C_max_ in the pediatric population PK analysis (11.6 pg/mL for FF 50 μg) [7].

By extrapolation, in the current study the predicted FF systemic exposure in terms of AUC was around 100 pg · h/mL. Established PK/PD models using nine studies of healthy subjects and adults and adolescents with asthma suggested that an AUC_0–24_ of 1000 pg h/mL would be required to reduce 24-h SC excretion by 20% and 24-h UC excretion by 17% [24]. Based on these data, no clinically relevant effects on cortisol levels were anticipated for the FF 50 μg dose, even in the smallest children and using the most conservative assumptions. The absence of an effect on serum cortisol in the present study confirmed these predictions, and based on these findings, FF 50 μg was anticipated to have an acceptable safety profile in children with persistent asthma aged 5–11 years.

The 24-h urine collection method is also reflective of cortisol production [18, 25] and it is widely used to assess HPA axis suppression in patients with asthma due to its non-invasive nature. However, UC measurements are mainly suitable for diagnosing oversecretion of cortisol (i.e., Cushing syndrome) rather than adrenal suppression for which assessment of SC is a more sensitive parameter. This is due to the variability of urine secretion as well as the fact that in adrenal suppression the lower levels of urinary free cortisol concentrations are not contributory to adrenal insufficiency [26].

In the present study, the FF 50 QD group had numerically lower UC levels compared with the placebo group. The ratio of FF 50 QD over placebo was 0.79 (95% CI 0.61, 1.03) for 24-h UC excretion and 0.88 (95% CI 0.72, 1.07) for 6-β hydroxycortisol excretion. Given the caveats associated with UC for the diagnosis of adrenal suppression, these results should not override the findings from SC measurements, which showed little difference between the FF 50 QD group and placebo. Furthermore, in this study, urine samples were only collected for 24-h rather than for 3 successive days, and the study was not statistically powered for assessing UC. However, as with all ICS, prescribers should remain vigilant in children treated with long-term FF.

Overall, FF 50 QD was well tolerated with no reported SAEs (fatal or non-fatal) or AEs leading to study withdrawal. There were no AEs consistent with adrenal insufficiency, and none of the three AEs of special interest reported were deemed related to study drug.

Strengths of the present study include the use of a robust methodology for assessing HPA axis function, high levels of exposure and compliance to study treatment, and a high number of complete 24-h profiles available. Domiciled clinic visits at the beginning and end of treatment were incorporated to standardize, control, and monitor the collection of blood and plasma parameters over 24 h. As part of the study protocol, peak SC values were captured in the morning in all children. Subject compliance with blinded inhaled medication was assessed by reviewing the dose counter on the inhaler.

The study has limitations. The 24-h SC concentration–time curve is not a good assessment of adrenal gland stress response, which should be measured using a dynamic function test such as the ACTH or the insulin tolerance test [27]. However, the gold standard insulin tolerance test is not usually carried out in pediatric patients due to safety considerations. The high-dose ACTH test is highly sensitive, although a positive result does not fully exclude the risk of adrenal failure. The low-dose ACTH test is more commonly used/preferred in clinical practice and is considered reliable for assessing the risk of adrenal crisis [28].

In addition, this study did not include an OCS (such as prednisolone) as a positive control due to ethical concerns related to corticosteroid administration in children younger than 12 years old at doses high enough to cause HPA axis suppression. This is consistent with other similar trials of ICS and intranasal corticosteroids in children. High peak SC levels may be explained by the study conditions (inpatient stay in a non-familiar setting) combined with the stress related to blood sampling in young children. Furthermore, the number of blood samples was limited in young children to assess more precisely the 24-h diurnal profile. Nevertheless, 24-h SC profiles were usually consistent between periods for individual subjects, with no cases of individual profiles showing a significant decrease in cortisol secretion at 6 weeks after treatment with FF by comparison with the baseline.

## Conclusions

The results of this study indicate that 6 weeks of treatment with the therapeutic pediatric dose of FF 50 QD was non-inferior to placebo on the HPA axis function, as assessed by weighted mean 24-h SC production. These data do not show evidence of a clinically significant suppressive effect of FF 50 QD on the HPA axis after 6 weeks of treatment in children aged 5–11 years. However, given the differences in sensitivity to ICS between patients, prescribers should remain vigilant in regard to adrenal function, especially in young children on long-term treatment with FF.

## Supplementary information

**Additional file 1.** Additional methods.

**Additional file 2: Figure S1.** Subject disposition.

**Additional file 3: Table S1.** Derived SC weighted mean (0–24 h) (SC and ITT population), geometric mean SC AUC_0–24_ (SC population), geometric mean 24-h UC excretion (UC population), and geometric mean 6-β hydroxycortisol excretion (UC population).

**Additional file 4: Table S2.** Change from baseline in peak SC (post hoc summary) and trough SC (24-h planned time point) at week 6 (SC population).

## Data Availability

Anonymized individual participant data and study documents can be requested for further research from http://www.clinicalstudydatarequest.com.

## Abbreviations

ACTH: adrenocorticotropic hormone
AE: adverse event
ANCOVA: analysis of covariance
AUC: area under the curve
CI: confidence interval
C_max_: maximum concentration
CV: coefficient of variation
FF: fluticasone furoate
FF 50 QD: FF 50 µg once daily
GINA: Global Initiative for Asthma
GCP: Good Clinical Practice
h: hour
HPA: hypothalamic–pituitary–adrenocortical
HPLC: high-performance liquid chromatography
ICH: International Conference for Harmonisation of Technical Requirements for Registration of Pharmaceuticals for Human Use
ICS: inhaled corticosteroid
ITT: intent-to-treat
LLQ: lower limit of quantification
LS: least squares
Max.: maximum
Min.: minimum
MS/MS: tandem mass spectrometer
OCS: oral corticosteroid
PD: pharmacodynamic
PK: pharmacokinetic
QD: once daily
SABA: short-acting β_2_-agonist
SAE: serious adverse event
SC: serum cortisol
SD: standard deviation
t_max_: time to peak plasma concentration
UC: urinary cortisol
VI: vilanterol
