# Supplementary material for: A randomized, double-blind, placebo-controlled, parallel-group study of once-daily inhaled fluticasone furoate on the hypothalamic–pituitary–adrenocortical axis of children with asthma
Source: Allergy Asthma Clin Immunol. 2020 Feb 4;16:11. doi: 10.1186/s13223-020-0406-6 (PMC7001316; doi:10.1186/s13223-020-0406-6)
Supplement: Supplementary file 1 — Additional file 1. Additional methods. [file 13223_2020_406_MOESM1_ESM.docx]

**Additional File 1**

**Additional Methods**

***Study Population***

Subjects were excluded if they had a history of life-threatening asthma or asthma exacerbation, defined as a deterioration of asthma requiring the use of systemic corticosteroids for at least 3 days; a depot corticosteroid injection within 3 months prior to screening; or hospitalization in the 6 months before screening; or had morning cortisol levels <2 µg/dL. Children were not eligible if they had received any of the following prior to Visit 1 and during the study: systemic, oral, or depot corticosteroids (within 12 weeks); inhaled corticosteroids or dermatological/topical corticosteroids (within 8 weeks); or intranasal corticosteroids (within 4 weeks). In addition, subjects were not permitted to use prescription or over-the-counter medications with potential to affect the course of asthma or the hypothalamic-pituitary-adrenocortical axis system.

Subjects were not eligible to enter the treatment period of the study if their asthma medication changed after Visit 1 and prior to randomization at Visit 2, if they had morning cortisol levels <2 μg/dL, anemia, or reduced peak expiratory flow levels.

Criteria for treatment withdrawal included severe asthma exacerbation, abnormal liver test or electrocardiogram results, or onset of menarche or pregnancy.

***Pharmacodynamic Assessments***

Urine cortisol and serum cortisol (SC) concentrations were determined using a high-performance liquid chromatography system (HPLC) by Cohesive Technologies in combination with a tandem mass spectrometer (LC/MS/MS) by Thermo Finnigan. This approach was designed to enhance specificity, reduce run-time, and simplify sample preparation.

***Populations and Statistical Analysis***

The sample size calculation was based on the primary endpoint. Approximately 100 subjects were to be randomized to complete at least 82 subjects in anticipation of a drop-out rate of approximately 10% by week 6 and of an additional 8% of subjects not meeting the SC per-protocol population criteria. Subjects were randomized to FF 50 μg inhalation powder administered once daily (FF 50 QD) or placebo with a ratio of 1:1. Non-inferiority was demonstrated if the lower limit of the two-sided 95% confidence interval for the geometric mean ratio of FF 50 QD and placebo was greater than 0.80. Assuming a standard deviation of 0.28, this study had approximately 90% power to demonstrate non-inferiority if there was a true difference (geometric mean ratio) between the treatment groups of 0.98.

Secondary SC endpoints were analyzed using the SC population and in the same way as the primary endpoint; however, non-inferiority margins were not predefined for these endpoints.
