# Supplementary material for: A randomized, double-blind, placebo-controlled, parallel-group study of once-daily inhaled fluticasone furoate on the hypothalamic–pituitary–adrenocortical axis of children with asthma
Source: Allergy Asthma Clin Immunol. 2020 Feb 4;16:11. doi: 10.1186/s13223-020-0406-6 (PMC7001316; doi:10.1186/s13223-020-0406-6)
Supplement: Supplementary file 2 — Additional file 2: Figure S1. Subject disposition. [file 13223_2020_406_MOESM2_ESM.docx]

**Additional File 2**

**Fig. S1**: Subject disposition.


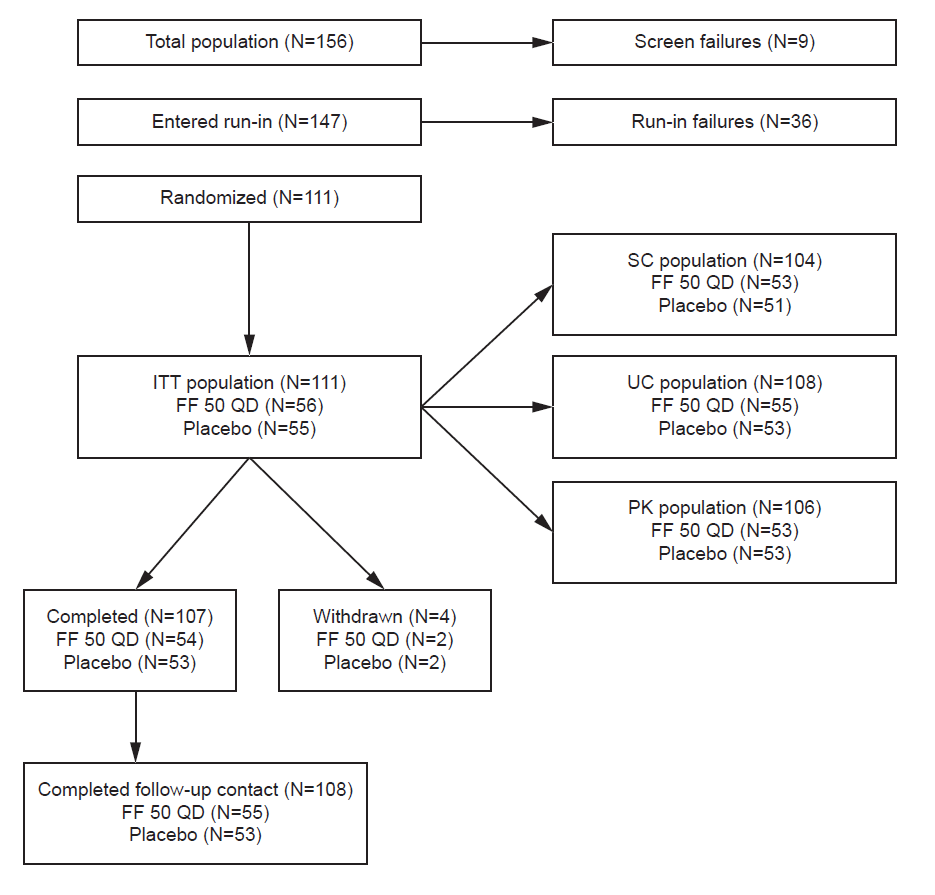


FF 50 QD, fluticasone furoate 50 µg once daily; ITT, intent-to-treat; PK, pharmacokinetic; SC; serum cortisol; UC, urinary cortisol.
