# Supplementary material for: A randomized, double-blind, placebo-controlled, parallel-group study of once-daily inhaled fluticasone furoate on the hypothalamic–pituitary–adrenocortical axis of children with asthma
Source: Allergy Asthma Clin Immunol. 2020 Feb 4;16:11. doi: 10.1186/s13223-020-0406-6 (PMC7001316; doi:10.1186/s13223-020-0406-6)
Supplement: Supplementary file 3 — Additional file 3: Table S1. Derived SC weighted mean (0–24 h) (SC and ITT population), geometric mean SC AUC0–24 (SC population), geometric mean 24-h UC excretion (UC population), and geometric mean 6-β hydroxycortisol excretion (UC population). [file 13223_2020_406_MOESM3_ESM.docx]

**Additional File 3**

**Table S1 Derived SC weighted mean (0-24 h) (SC and ITT population), geometric mean SC AUC_0–24_ (SC population), geometric mean 24-h UC excretion (UC population), and geometric mean 6-β hydroxycortisol excretion (UC population)**

| **Visit** | **Placebo  group** | **FF 50 QD  group** |
| --- | --- | --- |
| **SC population** |  |  |
| N | 51 | 53 |
| **Baseline** |  |  |
| n | 51 | 53 |
| Geometric SC weighted mean (0–24 h) (nmol/L) | 177.22 | 153.20 |
| SD logs | 0.319 | 0.344 |
| CV (%) | 32.70 | 35.43 |
| Median | 183.50 | 157.46 |
| Min., Max. | 97.2, 404.6 | 73.4, 345.6 |
| Geometric mean SC AUC_0–24_ (nmol/L) | 4248.65 | 3678.65 |
| SD logs | 0.318 | 0.343 |
| CV (%) | 32.63 | 35.34 |
| Median | 4404.00 | 3779.03 |
| Min., Max. | 2341.0, 9711.0 | 1761.0, 8294.0 |
| **Week 6** |  |  |
| n | 50 | 52 |
| Geometric SC weighted mean (0–24 h) (nmol/L) | 176.83 | 157.52 |
| SD logs | 0.368 | 0.364 |
| CV (%) | 38.14 | 37.68 |
| Median | 175.63 | 151.21 |
| Min., Max. | 67.5, 538.7 | 83.7, 519.1 |
| Geometric mean SC AUC_0–24_ (nmol/L) | 4251.31 | 3784.60 |
| SD logs | 0.370 | 0.364 |
| CV (%) | 38.30 | 37.65 |
| Median | 4215.00 | 3652.95 |
| Min., Max. | 1621.0, 12982.6 | 2008.0, 12475.4 |
| **Ratio from baseline** |  |  |
| n | 50 | 52 |
| Geometric SC weighted mean (0–24 h) (nmol/L) | 1.00 | 1.02 |
| SD logs | 0.427 | 0.448 |
| CV (%) | 44.77 | 47.16 |
| Median | 1.02 | 1.01 |
| Min., Max. | 0.3, 3.8 | 0.4, 4.1 |
| Geometric mean SC AUC_0–24_ (nmol/L) | 1.00 | 1.02 |
| SD logs | 0.427 | 0.448 |
| CV (%) | 44.73 | 47.11 |
| Median | 1.02 | 1.01 |
| Min., Max. | 0.3, 3.8 | 0.4, 4.1 |

| **ITT population** |  |  |
| --- | --- | --- |
| N | 55 | 56 |
| **Baseline** |  |  |
| n | 54 | 55 |
| Geometric SC weighted mean (0–24 h) (nmol/L) | 172.45 | 151.71 |
| SD logs | 0.359 | 0.343 |
| CV (%) | 37.14 | 35.30 |
| Median | 174.83 | 150.35 |
| Min., Max. | 46.5, 404.6 | 73.4, 345.6 |
| **Week 6** |  |  |
| n | 52 | 54 |
| Geometric SC weighted mean (0–24 h) (nmol/L) | 177.00 | 156.47 |
| SD logs | 0.366 | 0.360 |
| CV (%) | 37.83 | 37.17 |
| Median | 175.63 | 150.51 |
| Min., Max. | 67.5, 538.7 | 83.7, 519.1 |
| **Ratio from baseline** |  |  |
| n | 51 | 53 |
| Geometric SC weighted mean (0–24 h) (nmol/L) | 1.01 | 1.02 |
| SD logs | 0.428 | 0.444 |
| CV (%) | 44.79 | 46.71 |
| Median | 1.02 | 1.01 |
| Min., Max. | 0.3, 3.8 | 0.4, 4.1 |
| **UC population** |  |  |
| N | 53 | 55 |
| **Baseline** |  |  |
| n | 53 | 55 |
| Geometric mean 24-h UC excretion (nmol/24 h) | 58.27 | 74.73 |
| SD logs | 0.841 | 0.700 |
| CV (%) | 101.46 | 79.46 |
| Median | 60.80 | 69.90 |
| Min., Max. | 2.9, 247.9 | 24.1, 436.4 |
| n | 53 | 53 |
| Geometric mean 6-β hydroxycortisol excretion | 339.01 | 355.23 |
| SD logs | 0.495 | 0.585 |
| CV (%) | 52.68 | 63.86 |
| Median | 332.12 | 345.32 |
| Min., Max. | 120.0, 1113.7 | 95.4, 1730.7 |
| **Week 6** |  |  |
| n | 50 | 51 |
| Geometric mean 24-h UC excretion (nmol/24 h) | 61.53 | 51.51 |
| SD logs | 0.648 | 0.704 |
| CV (%) | 72.25 | 80.15 |
| Median | 62.20 | 47.30 |
| Min., Max. | 17.8, 484.1 | 8.5, 453.2 |
| n | 49 | 51 |
| Geometric mean 6-β hydroxycortisol excretion | 302.75 | 273.76 |
| SD logs | 0.494 | 0.522 |
| CV (%) | 52.54 | 56.02 |
| Median | 268.55 | 291.05 |
| Min., Max. | 119.1, 1012.2 | 83.4, 878.3 |
| **Ratio from baseline** |  |  |
| n | 50 | 51 |
| Geometric mean 24-h UC excretion (nmol/24 h) | 1.05 | 0.69 |
| SD logs | 0.964 | 0.772 |
| CV (%) | 123.86 | 90.20 |
| Median | 0.85 | 0.68 |
| Min., Max. | 0.2, 66.6 | 0.1, 11.6 |
| n | 49 | 50 |
| Geometric mean 6-β hydroxycortisol excretion | 0.90 | 0.78 |
| SD logs | 0.598 | 0.612 |
| CV (%) | 65.54 | 67.43 |
| Median | 0.89 | 0.79 |
| Min., Max. | 0.1, 4.3 | 0.1, 3.3 |

CV=100*sqrt(exp((SD logs)^2)-1), where SD logs denotes the SD of the logged values (or changes in logged values). N, number of subjects in population; n, number of subjects with value at the visit.

AUC_0–24_, area under the curve 0–24 h; CV, coefficient of variation; FF 50 QD, fluticasone furoate
50 µg once daily; h, hours; ITT, intent-to-treat; Min., minimum, Max., maximum; SC, serum cortisol; SD, standard deviation; UC, urinary cortisol.
