# Supplementary material for: A randomized, double-blind, placebo-controlled, parallel-group study of once-daily inhaled fluticasone furoate on the hypothalamic–pituitary–adrenocortical axis of children with asthma
Source: Allergy Asthma Clin Immunol. 2020 Feb 4;16:11. doi: 10.1186/s13223-020-0406-6 (PMC7001316; doi:10.1186/s13223-020-0406-6)
Supplement: Supplementary file 4 — Additional file 4: Table S2. Change from baseline in peak SC (post hoc summary) and trough SC (24-h planned time point) at week 6 (SC population). [file 13223_2020_406_MOESM4_ESM.docx]

**Additional File 4**

**Table S2 Change from baseline in peak SC (post hoc summary) and trough SC (24-h planned time point) at week 6 (SC population)**

| **Visit** | **Placebo** | **FF 50 QD** |
| --- | --- | --- |
| **SC population** |  |  |
| N | 51 | 53 |
| **Peak SC** |  |  |
| **Baseline** |  |  |
| n | 51 | 53 |
| Geometric mean peak SC (nmol/L) | 362.19 | 352.31 |
| SD logs | 0.378 | 0.357 |
| CV (%) | 39.22 | 36.86 |
| Median | 403.00 | 356.00 |
| Min., Max. | 168.0, 704.0 | 105.0, 657.0 |
| **Week 6** |  |  |
| n | 50 | 52 |
| Geometric mean peak SC (nmol/L) | 396.59 | 361.65 |
| SD logs | 0.332 | 0.362 |
| CV (%) | 34.18 | 37.37 |
| Median | 415.50 | 378.50 |
| Min., Max. | 188.0, 789.0 | 171.0, 811.0 |

| **Ratio from baseline** |  |  |
| --- | --- | --- |
| n | 50 | 52 |
| Geometric mean peak SC (nmol/L) | 1.10 | 1.03 |
| SD logs | 0.391 | 0.437 |
| CV (%) | 40.68 | 45.85 |
| Median | 1.05 | 1.04 |
| Min., Max. | 0.5, 3.2 | 0.3, 3.2 |
| **Trough SC** |  |  |
| **Baseline** |  |  |
| n | 51 | 53 |
| Geometric mean trough SC (nmol/L) | 290.56 | 271.86 |
| SD logs | 0.584 | 0.519 |
| CV (%) | 63.71 | 55.55 |
| Median | 339.00 | 282.00 |
| Min., Max. | 28.0, 629.0 | 36.0, 657.0 |
| **Week 6** |  |  |
| n | 50 | 52 |
| Geometric mean trough SC (nmol/L) | 296.64 | 315.51 |
| SD logs | 0.639 | 0.347 |
| CV (%) | 71.04 | 35.74 |
| Median | 325.50 | 313.00 |
| Min., Max. | 14.0, 660.0 | 132.0, 718.0 |

| **Ratio from baseline** |  |  |
| --- | --- | --- |
| n | 50 | 52 |
| Geometric mean trough SC (nmol/L) | 1.03 | 1.16 |
| SD logs | 0.801 | 0.560 |
| CV (%) | 94.91 | 60.74 |
| Median | 1.09 | 1.15 |
| Min., Max. | 0.0, 11.9 | 0.5, 11.3 |

CV=100*sqrt(exp((SD logs)^2)-1), where SD logs denotes the SD of the logged values (or changes in logged values). N, number of subjects in population; n, number of subjects with value at the visit.

CV, coefficient of variation; FF 50 QD, fluticasone furoate 50 µg once daily; h, hours; Min., minimum, Max., maximum; SC, serum cortisol; SD, standard deviation.
